# Preparation and study of two kinds of ophthalmic nano-preparations of everolimus

**DOI:** 10.1080/10717544.2019.1692966

**Published:** 2019-11-21

**Authors:** Zhan Tang, Lina Yin, Yawen Zhang, Wenying Yu, Qiao Wang, Zhajun Zhan

**Affiliations:** a College of Pharmaceutical Sciences, Zhejiang University of Technology, Hangzhou, PR China;; b Department of Pharmaceutics, Institute of Metaria Medica, Zhejiang Academy of Medical Sciences, Hangzhou, PR China;; c Key Laboratory of Neuropsychiatric Drug Research of Zhejiang Province, Institute of Materia Medica, Zhejiang Academy of Medical Sciences, Hangzhou, PR China

**Keywords:** Everolimus, micelles, nanosuspension, pharmacokinetics

## Abstract

*Objective:* To prepare everolimus nanoformulations and increase their solubility to suit their application in the eye. *Methods:* The everolimus micelles was prepared by thin film dispersion method using Tween-80 (P80) and polyoxyethylene stearate (P40S) as carriers. In addition, the everolimus nanosuspension was prepared by injection method using poloxamer 407 (P407), hydroxypropyl methylcellulose (HPMC) and polyvinyl alcohol (PVA) as stabilizers. It was characterized in terms of particle size, PDI and encapsulation efficiency or drug loading. The *in vitro* release and *in vitro* rabbit scleral permeability characteristics were investigated, and the pharmacokinetics of anterior chamber drug in rabbit eyes were studied. *Results:* The average particle size of the micelles was (8.74 ± 0.21) nm, the encapsulation efficiency and drug loading were (90.12 ± 1.18)% and (2.14 ± 0.028)%, while the average particle size of the nanosuspension was (156.47 ± 1.10) nm, and the drug loading was (16.51 ± 0.21)%, respectively. Both *in vitro* release and rabbit scleral permeation models were consistent with the Higuchi equation. The pharmacokinetic experiments of aqueous humor showed that area under the curve of everolimus nanosuspension was about 3 times higher than that of micelles. Micelles could be achieved in the eye and maintained for a long time. *Conclusion:* The preparation of everolimus micelles or nanosuspension for eye are suitable for ocular administration and expected to be new dosage form for corneal transplantation immunological rejection or other ocular disease.

## 1. Introduction

Everolimus, 4 *O*-*O*-(2-hydroxyethyl)-rapamycin, a hydroxy derivative of rapamycin, is a lipophilic 31-membered ring lactone compound isolated from Streptomyces hygroscopicus (Schuler et al., 1997; Kasper et al., 2018). In recent years, it has been found to specifically inhibit helper T lymphocytes, and has anti-immuno-rejection and anti-tumor activities (Herrera-Gómez et al., 2017; Huijts et al., 2017; Li et al., 2018; Wu et al., 2019). Studies have shown that it can be used in eyes such as autoimmune uveoretinitis, noninfectious uveitis, corneal neovascularization and immune-mediated rejection after corneal transplantation (Li et al., 2008; Çakmak et al., 2016; Blair et al., 2017; Kasper et al., 2018). Clinically, immune rejection diseases caused by organ transplantation including corneal transplantation are mostly treated by systemic administration such as oral administration, which is easy to cause systemic immunosuppressive effects and cause serious adverse reactions (Cotovio et al., 2012; Renner et al., 2012; Jebali et al., 2017). Especially for corneal transplantation, due to the presence of blood-eye barrier (Thiel et al., 2003), if a systemic administration is used, a large dose of oral administration is required to be effective in the eye, which will undoubtedly increase the risk of side effects such as thrombocytopenia and dyslipidemia which can occur in about 30% of the patients that take the drug (Baspinar et al., 2008; Tedesco-Silva et al., 2019). In contrast, the topical preparation of the eye has the advantages of small dosage and mild side effects, so it has become a hot spot for the development of clinical treatment drugs for corneal transplantation (Kauss Hornecker et al., 2015).

Nano-formulations have attracted extensive attention in the study of ocular administration due to their advantages of increasing drug solubility, improving drug bioavailability, slow/controlled release drug release (Zhao et al., 2018). The preparation of everolimus nano preparations such as nanoparticle (Seok et al., 2016), liposome (Iwase & Maitani, 2012), polymeric micelles (Rao et al., 2015), etc. had been reported in related literatures. It was hard to apply to practical industry situation because most involve the use of carrier materials that have potential irritating and toxic side effects on ocular tissues. Based on this, the research decided to use Tween-80 (P80) and polyethylene glycol stearate (P40S), which have good biocompatibility and have been approved by the US FDA for using as ophthalmic excipients, as carrier materials for micelles. In addition, PVP, HPMC and P407 are used as carrier materials for nanosuspensions. The micelles are self-assembled by diblock or multiblock amphiphilic molecules in the aqueous phase. The core-shell structure nanocarriers are thermodynamically stable. The homogeneous solution, the micellar preparation can encapsulate the poorly soluble drugs, which has the advantages of improving the solubility of the drug and alleviating adverse reactions (Li et al., 2019; Liu et al., 2019; Shi et al., 2019). This experiment investigated the possibility of making everolimus into an ophthalmic micellar and nanosuspension preparation, providing a basis for its subsequent application development in the eye.

## 2. Materials and methods

### 2.1. Materials

Everolimus (EVR, purity > 98%) was purchased from Lunan Pharmaceutical Group Corporation (Shandong, China), and internal standard ascomycin (IS) was purchased from Gene Operation (Ann Arbor, MI, USA, purity > 99%, QAS1212). Tween-80 and polyoxyethylene (40) stearate (P40S) were supplied by Sigma-Aldrich (St. Louis, MO, USA). Pluronic P407 was from BASF (Ludwigshafen, Germany). Hydroxypropylmethylcellulose and glutamine were purchased from Aladdin (Shanghai, China). Polyvinyl alcohol with low viscosity was obtained from Jiangxi Alpha Gaoke Pharmaceutical Corporation (Jiangxi, China). Methanol was purchased from Merk (Darmstadt, Germany), acetonitrile was from Tedia (Fairfield, OH, USA), and formic acid was from ACS (Wilmington, DE, USA) and all were of HPLC-grade. Ammonium formate was supplied by Sinopharm Chemical Reagent Co., Ltd (Shanghai, China). Water purification was carried out with the Milli-Q ultra-pure water system (Millipore, Darmstadt, Germany). Other reagents were of analytical grade and commercially available.

New Zealand albino rabbits (2.5–3.0 kg) were purchased from Zhejiang Laboratory Animal Center (Hangzhou, China). Rabbits were bred under ambient temperatures of about 20 °C and were adapted to the environment for at least one week before use. Standard laboratory food and water were freely accessible to all animals until 12 h prior to the experiments, at which time only water was given. All experiments were conducted according to the animal care and use guidelines established by the Zhejiang Academy of Medical Sciences. All animal experiments were approved by the animal ethics/use committee of Zhejiang Academy of Medical Sciences.

### 2.2. Preparation of everolimus micelles and nanosuspension

Everolimus micelles was prepared by thin-film hydration method. Briefly, P40S (0.02 g) and everolimus (5 mg) were dissolved in 2.5 mL of 95% ethanol, the solution was evaporated in a rotator evaporator at 100 rpm and 40 °C to remove the alcohol. Subsequently, 5 mL of prewarmed distilled water with Tween-80 (0.18 g) was added, the film dissolved in the water phase by hand shaking and ultrasonic dispersion. To obtain the micelles, the sample was subjected to centrifugation at 10,000 rpm for 10 min to remove the insoluble substances. Supernatant was stored in vial bottle at 4 °C for further application or characterization.

Everolimus nanosuspension was prepared by injection method. Briefly, PVA (0.0298 g) and HPMC (0.0106 g) were mixed with 10 mL distilled water and heated, stirring until the solution was completely dissolved. P407 (0.0073 g) and everolimus (10 mg) were dissolved in 150 μL of 95% ethanol. 150 μL of solution was injected into 10 mL of solution and magnetically stirred for 10 min. The nanosuspension exhibited a pale blue opalescent appearance and was stored in vial bottle at 4 °C for further application or characterization.

### 2.3. Osmotic pressure and pH

Ophthalmic preparations are required to be isotonic with ocular surface tears (280 ∼ 310 mOsm·L^−1^), and the pH of the eye drops is generally in the range of 5.5 ∼ 7.8 to avoid irritation to the eye tissue. The osmotic pressure of micelles and nanosuspension were determined by a freezing point osmometer (STY-1, Tianjing, China). Mannitol or 1,2-propanediol were used as an osmotic pressure adjusting agent. The pH value was determined by a precision pH meter (PHS-3C, Shanghai, China), and 0.2% tromethamine was used as a pH adjuster.

### 2.4. Particle size, PDI and zeta potential

One milliliter of the samples without any dilution were taken at room temperature, placed in the disposable sizing cuvettes or fold capillary cells, and the particle size distribution, polydispersity (PDI), and zeta potential were measured by a Zetasizer (Nano-ZS90, Malvern Instruments, Worcestershire, UK), and repeated three times.

### 2.5. Transmission electron microscopy

Everolimus loaded in micelles or nanosuspension were visualized with JEM-1200EX (JEOL Co., Ltd., Tokyo, Japan). The micelles sample was diluted 10 times (or the nanosuspension sample was taken directly), and the appropriate amount was taken on the copper mesh. After the excess solution was carefully absorbed by the filter paper, the samples were stained with uranyl acetate, and the surface moisture was again absorbed by the filter paper, and dried at room temperature.

### 2.6. Drug loading (DL) and entrapment efficiency (EE)

High performance liquid chromatography conditions: the HPLC system was Shimadazu (Kyoto, Japan) equipped with a UV detector (SPD-20A) set at 277 nm and binary pumps (model LC-20AD). The separation was performed on a C18 column (Diamonsil, Beijing, China, 4.6 mm × 150 mm, 5 μm) with acetonitrile-water (86:14) as mobile phase under gradient elution at a flow rate of 1.0 mL·min^−1^. The column temperature was set at 60 °C and injection volume was 20 μL.

Everolimus micelles sample was subjected to centrifugation at 10,000 rpm for 10 min to remove the insoluble substances. So the EE was measured as follows: Briefly, 100 μL of the micelles was placed in a 7 mL centrifuge tube, 3.9 mL of acetonitrile was added, and followed by 10 min ultrasound then centrifuged at 10,000 rpm for 5 min to obtain the supernatant. The amount of everolimus in the resulting solution analyzed by HPLC was labled W1. Each sample was measured three times.

The DL was calculated using the following equations: Briefly, 100 μL prepared nanosuspension was mixed with 3.9 mL acetonitrile and followed by 10 min ultrasound then centrifuged at 10,000 rpm for 5 min to obtain the supernatant. The amount of everolimus in the resulting solution analyzed by HPLC was labled W2. Each sample was measured three times.

$$\text{EE}(\%)=\frac{W1}{\text{Weight}\text{of}\text{the}\text{fed}\text{drug}}\times 100$$

$$\text{DL}(\%)=\frac{W1\text{or}W2}{\text{Weight}\text{of}\text{the}\text{drug}\text{and}\text{excipient}}\times 100$$

### 2.7. *In vitro* drug release studies

Preparation of *in vitro* release standard curve: 40% PEG-phosphate buffer solution (pH 7.4) was used as medium to prepare a series of standard solutions of everolimus mass concentration at 0.05, 0.09, 0.19, 0.37, 0.75, 1.50, 3.00, 5.99, and 11.99 μg·mL^−1^.

*In vitro* release study was performed for micelles and nanosuspension using dialysis techinique (Fangueiro et al., 2016; Jin et al., 2018). 0.5 mL above formulations were placed in dialysis bag (Mw 14000) and tightly sealed. The release medium was phosphate buffer saline of pH 7.4 containing 40% PEG. The dialysis bags were then immersed in release medium, then put them into shaker (34 ± 0.5 °C, 100 rpm·min^−1^). The 1 mL of samples were withdrawn and replaced with the same volume of fresh medium at predetermined time points (2, 4, 6, 8, 10, 24, 48, 72, 96 h). The samples (1 mL) were centrifuged at 10,000 rpm for 5 min and analyzed by HPLC. The cumulative release rate (*Q_n_*) was calculated according to the following formula (Equation 1), the cumulative release versus time curve was plotted, and the release curves were fitted using the Origin 8.0 software.

(1) $$Q_{n}=\frac{V_{0}C_{n}+V\sum_{i=1}^{n-1}C_{i}}{m_{\text{drug}}}$$

where *Q_n_* is the cumulative release rate or cumulative permeability (%), *V* is the sampled volume (mL), *V*_0_ is the total volume (mL) of the release or receiving medium solution, and *C_n_* is the drug concentration measured at time *t* (mg·mL^−1^), *C_i_* is the concentration measured before *t* time (mg·mL^−1^), and *m*_drug_ is the total mass (mg) of everolimus in the micelles or nanosuspension.

In order to study the mechanism of release of everolimus from nanoparticles (micelles and nanosuspension), in vitro release data were analyzed by using the following different kinetic models (Yarce et al., 2016; Akbari & Wu, 2017; Guan et al., 2019):

Zero-order equation

$$Q_{t}=Q_{0}+K_{0}t$$

where *Q_t_* is the amount of the drug released at time *t*, *Q*_0_ is the initial amount of drug in the solution, and *K*_0_ is zero-order release constant.

First-order equation

$$Q_{t}=a\left[1-\;\text{exp}\;(-bt)\right]$$

where *Q_t_* is the amount of the drug released at time *t*, a and b are the first order rate constants.

Higuchi equation

$$Q_{t}=K_{H}t^{1/2}+b$$

where *Q_t_* is the amount of the drug released at time *t*, *K*_H_ and *b* are the Higuchi constant.

The obtained results were statistically analyzed, and correlation coefficient *R*^2^ was calculated using Origin 8.0.

### 2.8. Corneal and scleral permeation studies in rabbit eyes

Cornea and scleral used in the experiment were obtained from New Zealand white rabbits eyes with no trauma and sacrificed by air needles through ear veins. The rabbit eyeballs were carefully taken with surgical scissors and forceps, and the eyeball tissue was separated, and muscles and nerves on the surface of the cornea or sclera were removed, then the intact and uninjured sclera was washed with saline several times and immediately used for penetration experiments.

Referring to the relevant literature (Punyamurthula et al., 2017; Mo et al., 2018; Sayed et al., 2018), the rabbit corneal and scleral penetration experiment of everolimus micelles and nanosuspension were carried out using a self-made curved Franz diffusion cells. A freshly configured 40% PEG-phosphate buffer was added to the acceptor cell as the receiving medium, and the fresh excised cornea or sclera was then fixed between the donor and acceptor compartments of the Franz diffusion cells, with the outer layer of the cornea or sclera facing the donor cell. The water bath cycle temperature of the acceptor pool was set at 34 ± 0.5 °C, the magneton speed was 100 r·min^−1^. The opening of the receiving pool is sealed with a sealing film to prevent the evaporation of permeate. Finally, 80 μL of the prepared everolimus micelles or nanosuspension were added to the dornor compartments (*n* = 6). 100 μL of samples were taken from the acceptor cell at 10, 24, 48, 72, and 96 h, respectively, and an isothermal equal volume acceptor medium solutions were added. After the samples were centrifuged (10,000 rpm, 5 min), the supernatant were taken for HPLC analysis. The cumulative drug permeation rate (*Q_n_*) was calculated according to the formula (Equation 1), and the cumulative penetration versus time curve was plotted, and the permeation curve was fitted using the Origin 8.0 software.

### 2.9. Short-term stability

The prepared samples were sealed in glass vials and stored at 4 °C, 40 °C and light (4500 lx ± 500 lx). After preparation (Day 0), 5, 10, 30, 60, 90, and 180 day, the sample was evaluated for DL% and EE% according to the above method.

### 2.10. Pharmacokinetics studies in rabbit eyes

The HPLC system comprised of an Agilent1290 HPLC equipped with a G4204A pump, G4226A autosampler, and a detector G6460A (Waltham Massachusetts United-States). All chromatograms were recorded using the MassHunter workstation software (Agilent Technologies Inc., USA). The stationary phase was reverse-phased phenyl C18 (4.6 × 150 mm, 3.5 µm, Waters Xbridge™, USA) column. The mobile phase consisted of water (containing 5 mM ammonium formate and 0.1% formic acid) (A) and methanol (containing 0.1% formic acid) (B) (10:90, V:V) at a flow rate of 0.6 mL/min. The column oven was set at 50 °C. The injection volume was 20 μL.

Everolimus and ascomycin were monitored by a triple quadrupole 6460 mass spectrometer (Agilent Technologies Inc., USA) with an electrospray ionization source (ESI). Multiple reaction monitoring (MRM) with positive ionization mode was used for the mass analysis. MS conditions were set as follows: capillary voltage 3500 V; nozzle voltage 500 V, and nebulizer gas (nitrogen) pressure 45 psi. Gas temperature was set at 300 °C and gas flow at a rate of 5 L·min^−1^. Sheath gas flow rate was set at 11 L·min^−1^ with the temperature of 250 °C. The m/z of ion pairs used for monitoring were 975.4 → 908.4 for everolimus and 809.4 → 756.5 for ascomycin (IS). The collision energy and fragmentor voltage were 16.0 V and 135 V for everolimus, 20.0 V and 150.0 V for IS, respectively. Cell accelerator voltage for the drug and IS were 7.0 V. The quantitative analysis of everolimus in rabbit eye aqueous humor was based on the peak area ratio (peak area of the drug versus peak area of IS).The mass spectrometry parameters are shown in Table 1.

**Table 1.** MRM parameters for compounds.

Compounds	Parent ion/fragment ion (m/z)	Fragmentor (V)	Collision energy (V)	Cell accelerator voltage (V)
Everolimus	975.4/908.4	135	16	7
IS	809.4/756.5	150	20	7

Ten healthy New Zealand albino rabbits weighing 2.5–3.0 kg were randomly divided into micelles group and nanosuspension group (5 animals for each group) that were kept for 1 week before the experiment. Animals were anesthetized with 25% urethane (1 g/kg) throughout the experiment. 1% tropicamide was dropped into the eye to enlarge the pupil and the eyelid was opened with a sputum. A 29 G needle was inserted from the cornea end edge and medical adhesives were used to close the needle hole. A soft silicone tube 8 cm (ID 0.2 mm × OD 0.5 mm) was attached to the tail of the needle. The silicone tube was clamped with an artery clamp, which was opened to release the aqueous humor samples. A single dosage (80 μL) of 1 mg/mL everolimus (micelles or nanosuspension) eye drops was instilled into the conjunctival sac of the eye. Approximately 25–30 μL of the aqueous humor samples were collected at 0, 0.5, 1, 2, 3, 4, 5, 6, 7, 8, 9, 10 h and were stored at −70 °C until analysis. The pharmacokinetic parameters were assessed using DAS VER 2.0 software (Mathematical Pharmacology Professional Committee of China, Shanghai, China). Pharmacokinetic parameters were calculated using a non-compartmental analysis with a linear trapezoidal model.

## 3. Results and discussion

### 3.1. Characterization of micelles and nanosuspension

**Figure 1.**
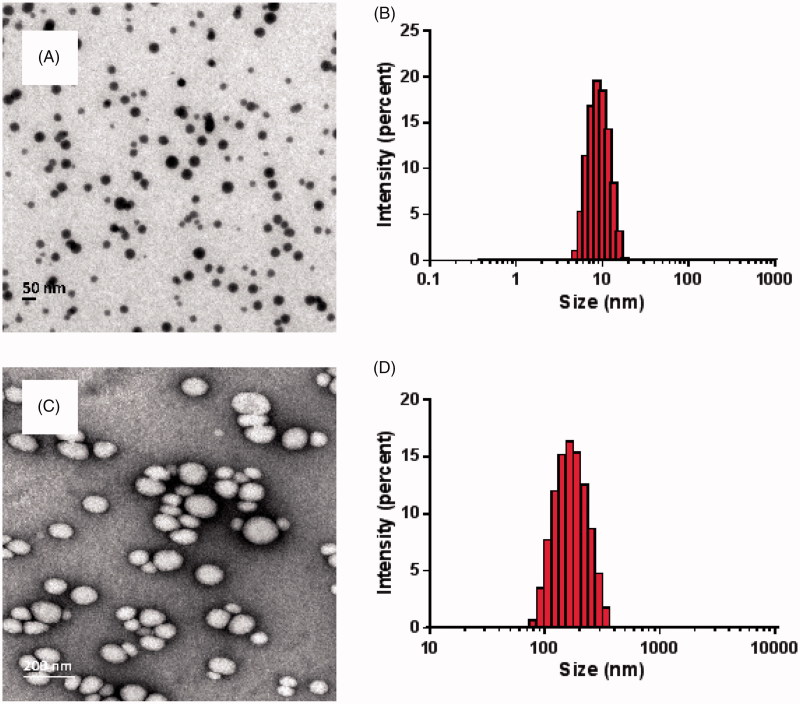
Morphology (A) and size distribution (B) of the everolimus micelles; Morphology (C) and size distribution (D) of the everolimus nanosuspension.

The particle size of the everolimus micelles and nanosuspension were 8.74 ± 0.21 nm and 156.47 ± 1.10 nm, respectively. As shown in Figure 1, the particle size showed a single peak distribution, the zeta potential were just −2.12 mV and −8.99 mV, close to 0 mV, and the polydispersity coefficient PDI were 0.067 ± 0.012 and 0.091 ± 0.005, indicating good dispersion. The Zeta potential was used as an indicator of physical stability in a colloidal system, and its absolute value above 30 was considered to be physically stable (Freitas & Müller, 1998). However, in the case of using a steric stabilizer, the Zeta potential is used as an indicator of surface coverage (Verma et al., 2009; Seok et al., 2016). Therefore, a decrease in the absolute Zeta potential of the surface coverage of the drug particles means a physically stable state rather than an unstable state. There is no correlation between the particle size of the space stabilizer and the zeta potential (Verma et al., 2009; Seok et al., 2016). The smaller size observed in TEM images may be due to the drying effect during sample preparation for TEM measurements compared to dynamic light scattering. This phenomenon was also found in other nanoformulation sample (Shen et al., 2018). The EE% and DL% of the everolimus micelles formulation were 90.12 ± 1.18% and 2.14 ± 0.028%, respectively. And DL of nanosuspension was 16.51 ± 0.21%. The solubility of everolimus as a raw material at room temperature was about 9.6 μg·mL^−1^ (Iwase & Maitani, 2012), so this experiment successfully increased the solubility of everolimus by 104 times.

### 3.2. *In vitro* drug release

The standard curve regression equation was obtained by using 40% PEG-phosphate buffer as media: *Y* = 53656.642*X* − 652.217, *R*^2^=0.999 (*n* = 5), indicating the concentration range of 0.0 5 ∼ 11.99 μg·mL^−1^ has a good linear relationship. And the specificity, precision, accuracy, stability, etc. were investigated, and the results were all in line with the requirements.

**Figure 2.**
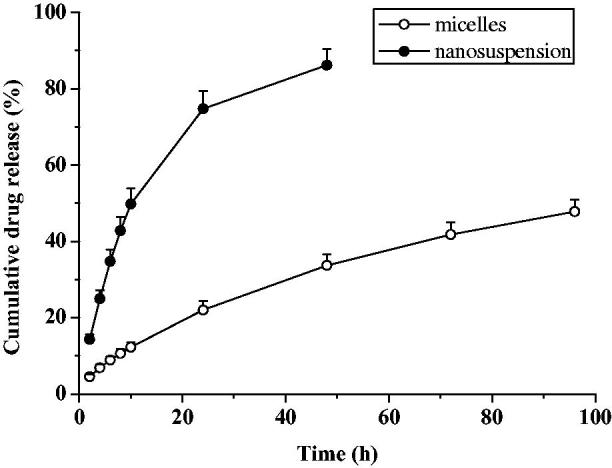
*In vitro* cumulative release profile (*n* = 6, mean ± SD).

The cumulative release profile of everolimus micellar and nanosuspension preparation were shown in Figure 2. It could be seen from the figure that the micellar preparation continuously released within 96 h, and the cumulative release at 96 h reached 51.97%, and there was still sustained release trend. The cumulative release rate of micelles at 96 h was only 47.74%, but there was still a trend of sustained release. It can be seen that the micelles have a significant sustained release effect compared to the suspension. Only 22.00% of everolimus was released into the medium within 24 h; while the suspension release rate was 74.69% in 24 h, which is 3.4 times that of micelles. However, after release of 86.15% of the suspension for 48 h, the release was in the plateau and the drug was no longer released.

The zero-order kinetics, first-order kinetics and Higuchi equation were used to fit them respectively. The fitting results were shown in Table 2. It could be seen that the Higuchi and first-order equation had the best fitting effect for micelles and nanosuspension, *R*^2^ were 0.9986 and 0.9995, respectively.

**Table 2.** *In vitro* release fitting parameters of different models.

Formulation	Models	Equations	*R*^2^
Micelles	Zero-order	*Q* = 0.508*t* + 5.117	0.9462
	First-order	*Q* = 50.664(1 − e^−0.025^*^t^*)	0.9884
	Higuchi	*Q* = 5.324×(*t*^0.5^)−3.916	0.9986
Nanosuspension	Zero-order	*Q* = 1.728*t* + 16.483	0.7988
	First-order	*Q* = 87.140(1 − e^−0.0843^*^t^*)	0.9995
	Higuchi	*Q* = 13.207×(*t*^0.5^)+2.066	0.9348

Since Higuchi’s model follows pure diffusion-controlled release (Fick diffusion) (Akbari & Wu, 2017), and the micelles agree well with this equation, it can be speculated that the drug release mechanism of micelle particles in 40% PEG-phosphate buffer was based on diffusion-release. In addition, the release of the nanosuspension was faster than that of the micelles.

### 3.3. Corneal and sclera permeation studies in rabbit eyes

**Figure 3.**
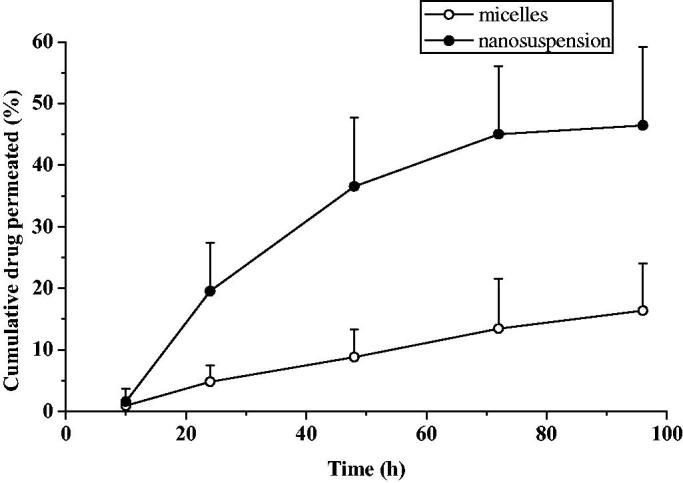
Cumulative penetration profile of isolated rabbit sclera (*n* = 6, mean ± SD).

**Figure 4.**
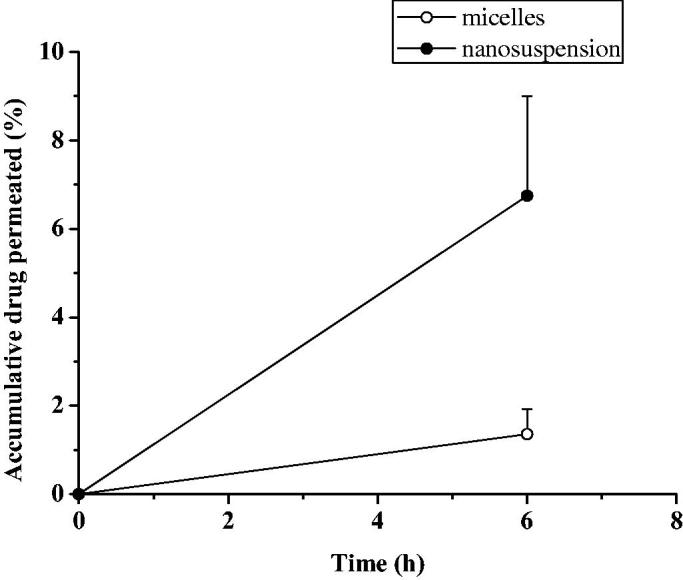
Cumulative penetration profile of isolated rabbit cornea (*n* = 3, mean ± SD).

The accumulation curve of rabbit sclera or cornea in everolimus micellar and nanosuspension preparation were shown in Figures 3 and 4. Similar to the *in vitro* release results, it can be found that the micellar preparation can continue to penetrate continuously within 96 h, and the cumulative penetration at 96 h reaches 17.6% in sclera. And had a tendency to sustain release. The zero-level kinetics, first-order kinetics, and Higuchi equation were used to fit the infiltration process. The fitting results were shown in Table 3. It can be seen that the Higuchi equation had the best fitting effect, and *R*^2^ were 0.9958 and 0.9540, respectively. This was consistent with the results of *in vitro* release, and the penetration of everolimus micelles into the sclera was also carried out by fick diffusion. In vitro corneal cumulative penetration of everolimus for micelles and nanosuspension were 1.36 ± 0.56% and 6.75 ± 2.25% in 6 h, respectively. Preparation of everolimus nanosuspension increased corneal permeability of the drug, with cumulative amounts of drug permeated after 6 h increased 5-fold, compared with micelles. The particle size of nanosuspensions were significantly higher than micelles while the penetration of nanosuspension was more than micelles. One possible reason was that everolimus was extremely difficult to dissolve in water. When everolimus was coated in the hydrophobic core of the micelles, the concentration of the drug in contact with the cornea or sclera was low, and the amount of penetration into the cornea or sclera from the concentration gradient was relatively low. While the nanosuspension particles were relatively large, without the outer layer of the micelles, the concentration of the drug in contact with the cornea or sclera was high, and the amount of penetration according to the concentration gradient was high.

**Table 3.** Isolated rabbit scleral penetration fitting parameters of different models.

Formulation	Models	Equations	*R*^2^
Micelles	Zero-order	*Q* = 0.184*t* − 0.485	0.9842
	First-order	*Q* = 104.12(1 − e^−0.00183^*^t^*)	0.9824
	Higuchi	*Q* = 2.311×(*t*^0.5^)−6.462	0.9958
Nanosuspension	Zero-order	*Q* = 0.652*t* − 4.444	0.9189
	First-order	*Q* = 68.955(1 − e^−0.0132^*^t^*)	0.9297
	Higuchi	*Q* = 7.147×(*t*^0.5^)−18.032	0.9540

### 3.4. Short-term physical stability

**Figure 5.**
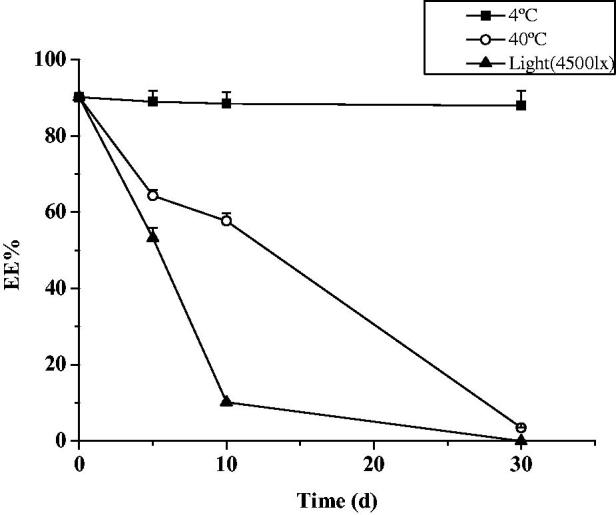
Micelle encapsulation rate change under different conditions (*n* = 3, mean ± SD).

**Figure 6.**
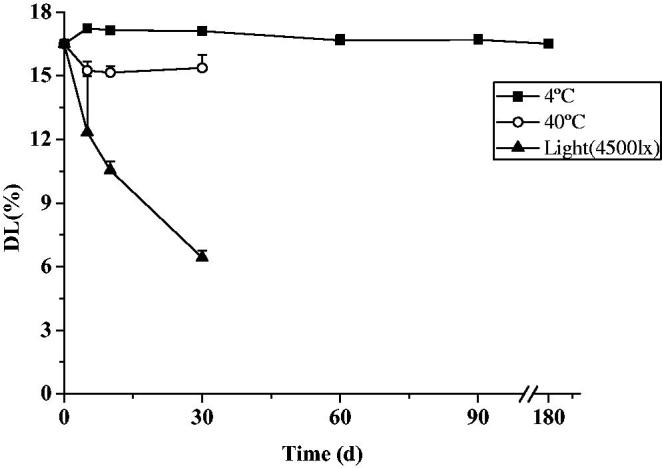
The drug loading of nanosuspension changes with time under different conditions (*n* = 3, mean ± SD).

Both everolimus micelles and nanosuspension exhibited good thermodynamic stability and very low drug leakage rates during storage under 4 °C. No visual precipitation was observed in the formulation. When storded at temperatures of 40 °C, the EE% and DL% decreased (Figures 5 and 6). The decrease in everolimus micelles EE% was significantly increased. Degradation was more obvious under lighting condition for both micelles and nanosuspension, the EE% of everolimus micelles changed from the initial 90.12 ± 1.18% to below the limit of detection for 30 d, while DL % of nanosuspension changed from the initial 16.51 ± 0.21% to 6.43 ± 0.33%. Therefore, 4 °C away from light was an advantageous storage condition for everolimus micelles and nanosuspension.

### 3.5. Pharmacokinetics studies in rabbit eyes

Prior to pharmacokinetic studies, HPLC-MS/MS was developed and validated for the quantitative determination of the concentration of everolimus in rabbit aqueous humor. Everolimus showed good linearity in the range of 1.5–500 ng/mL. The relationship was obtained by linear regression of the sample concentration (X) with the ratio of the peak area of the ion mass spectrum of the everolimus to the peak area of the corresponding internal standard ion mass spectrum (*X*). Linear regression was performed using a weighted (1/*X*^2^) least squares method. The relative standard deviation (RSD) of the intra- and inter-day precision of the four different concentrations (1.5, 2.5, 200, and 400 ng/mL) was <7.78%, and the accuracy ranged from 91.77% to 107.78%. The results showed that the HPLC-MS/MS method for the determination of everolimus with good sensitivity and reproducibility was suitable for pharmacokinetic studies.

**Figure 7.**
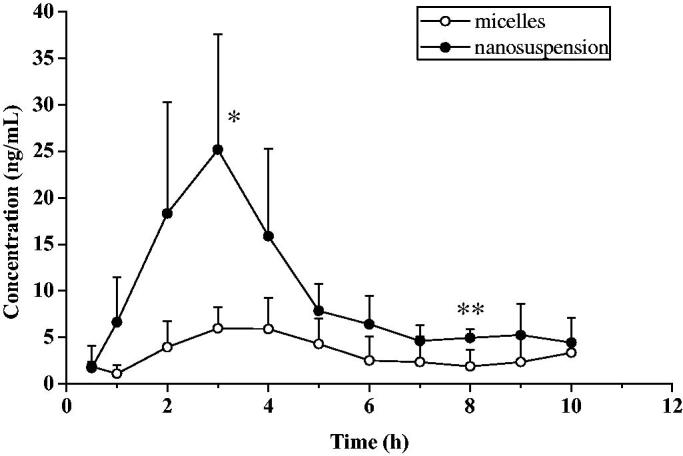
Mean aqueous humor concentration-time curve of everolimus after ocular administrations of micelles and nanosuspension (*n* = 5, mean ± SD).

The kinetic curve of the anterior chamber of rabbit eye of everolimus micelles and nanosuspension were shown in Figure 7. The main pharmacokinetic parameters were shown in Table 4. It can be seen that the ocular absorption of micelles was found to be lower than nanosuspension. The C_max_、T_1/2_ and AUC_(0–t)_ of the nanosuspension were approximately 3 times higher than the micelles. Nanosuspensions had a shorter T_max_ than micelles. The micellar preparation achieved T_max_ at 4.4 h with C_max_ of 8.4 ng/mL. While the nanosuspension reaches C_max_ in 2.6 h, and the C_max_ value was 27.0 ng/mL, which was significantly higher than that of micelles (*p*<0.05). AUC of everolimus from micelles was 32.274 ± 4.656 μg·h·L^−1^, and extremely significantly lower than that of nanosuspension with 95.709 ± 33.517 μg·h·L^−1^ (*p*<0.01). These results indicated that the nanosuspension-based formulation enhanced the permeability of everolimus through the cornea and was absorbed into the aqueous humor. In addition, the nanosuspension had a smaller clearance than the micelles with a significant difference (*p*<0.05), indicating that the nanosuspension can reduce the elimination of everolimus in the aqueous humor. The micelles exhibited sustained release characteristics consistent with the results of in vitro release and excision of the isolated sclera, indicating that the formulation can persist in the eye for a period of time.

**Table 4.** Main pharmacokinetic parameters of everolimus in rabbit aqueous humor after ocular administrations of micelles and nanosuspension (*n* = 5, mean ± SD).

Parameter		Micelles	Nanosuspension
AUC_(0–_*_t_*_)_	μg·h·L^−1^	32.274 ± 14.656	95.709 ± 33.517**
MRT_(0–_*_t_*_)_	H	4.598 ± 0.923	4.183 ± 0.509
*t*_1/2_	H	2.189 ± 1.139	6.346 ± 6.142
*T*_max_	H	4.400 ± 3.209	2.600 ± 0.548
CLz/F	L·h^−1^	2.462 ± 1.280	0.678 ± 0.267*
Vz/F	L	6.710 ± 2.651	4.558 ± 1.468
*C*_max_	μg·L^−1^	8.439 ± 2.274	27.017 ± 12.133*
AUC_nanosuspension_/AUC_micelles_		2.966	

* *p*<.05 micelles compared with nanosuspension.

** *p*<.01 micelles compared with nanosuspension.

## 4. Conclusion

In summary, a topical application of everolimus micellar formulations based on the nonionic surfactant Tween 80 was successfully developed. The micelles loaded with everolimus had a small droplet size, good physical stability and high encapsulation efficiency, while the everolimus nanosuspension was successfully obtained by injection method with low dose excipients. Compared with micelles, everolimus suspension had higher release, permeability and bioavailability. Micelles and nanosuspensions have great promise as effective carriers for everolimus in the treatment of ocular disease.
